# A High-Throughput *In Vitro* Drug Screen in a Genetically Engineered Mouse Model of Diffuse Intrinsic Pontine Glioma Identifies BMS-754807 as a Promising Therapeutic Agent

**DOI:** 10.1371/journal.pone.0118926

**Published:** 2015-03-06

**Authors:** Kyle G. Halvorson, Kelly L. Barton, Kristin Schroeder, Katherine L. Misuraca, Christine Hoeman, Alex Chung, Donna M. Crabtree, Francisco J. Cordero, Raj Singh, Ivan Spasojevic, Noah Berlow, Ranadip Pal, Oren J. Becher

**Affiliations:** 1 Department of Pediatrics, Duke University Medical Center, Durham, NC, United States of America; 2 Department of Pathology, Duke University Medical Center, Durham, NC, United States of America; 3 Preston Robert Tisch Brain Tumor Center, Duke University Medical Center, Durham, NC, United States of America; 4 Department of Surgery, Duke University, Durham, NC, United States of America; 5 Graduate Program in Molecular Cancer Biology, Duke University, Durham, NC, United States of America; 6 Department of Medicine—Oncology, Duke University, Durham, NC, United States of America; 7 Duke Cancer Institute, Pharmaceutical Services—PK/PD Core Laboratory, Durham, NC, United States of America; 8 Department of Electrical and Computer Engineering, Texas Tech University, Lubbock, TX, United States of America; University Hospital of Navarra, SPAIN

## Abstract

Diffuse intrinsic pontine gliomas (DIPGs) represent a particularly lethal type of pediatric brain cancer with no effective therapeutic options. Our laboratory has previously reported the development of genetically engineered DIPG mouse models using the RCAS/tv-a system, including a model driven by PDGF-B, H3.3K27M, and p53 loss. These models can serve as a platform in which to test novel therapeutics prior to the initiation of human clinical trials. In this study, an *in vitro* high-throughput drug screen as part of the DIPG preclinical consortium using cell-lines derived from our DIPG models identified BMS-754807 as a drug of interest in DIPG. BMS-754807 is a potent and reversible small molecule multi-kinase inhibitor with many targets including IGF-1R, IR, MET, TRKA, TRKB, AURKA, AURKB. *In vitro* evaluation showed significant cytotoxic effects with an IC_50_ of 0.13 μM, significant inhibition of proliferation at a concentration of 1.5 μM, as well as inhibition of AKT activation. Interestingly, IGF-1R signaling was absent in serum-free cultures from the PDGF-B; H3.3K27M; p53 deficient model suggesting that the antitumor activity of BMS-754807 in this model is independent of IGF-1R. *In vivo*, systemic administration of BMS-754807 to DIPG-bearing mice did not prolong survival. Pharmacokinetic analysis demonstrated that tumor tissue drug concentrations of BMS-754807 were well below the identified IC_50_, suggesting that inadequate drug delivery may limit *in vivo* efficacy. In summary, an unbiased *in vitro* drug screen identified BMS-754807 as a potential therapeutic agent in DIPG, but BMS-754807 treatment *in vivo* by systemic delivery did not significantly prolong survival of DIPG-bearing mice.

## Introduction

An estimated 4,000 new malignant and non-malignant brain tumors are diagnosed annually in children in the United States [1, 2]. Up to 20% of malignant central nervous system (CNS) tumors in children arise in the brainstem, with the majority being the diffuse intrinsic pontine glioma (DIPG) subtype [1, 3]. DIPG is a high-grade glioma (HGG) that originates in the pons and is seen almost exclusively in children with a median age at diagnosis of 6 to 7 years [4, 5].

Despite numerous clinical investigations, to date there are no clearly beneficial chemotherapeutic or biological agents for the treatment of DIPG. The standard treatment remains conventional focal radiotherapy (RT) of 54–60Gy, fractionated over a 6-week period [6]. This treatment has been shown to transiently improve neurologic function or temporarily stabilize disease in 70% of patients, but the effect on overall survival is minimal with more than 90% of children dying within two years of diagnosis [4–7].

A key to improving these outcomes is to gain a better understanding of DIPG tumor biology. In France, biopsies have been routinely performed since 2003 for both atypical and typical DIPG evaluation, and have been associated with minimal morbidity and high diagnostic yield [8]. This surgical success has also led to an increase in the availability of DIPG tumor samples, providing much of the new biologic, molecular, and genetic data that is currently advancing our understanding of DIPG. The most common genetic alterations include mutually exclusive K27M mutations in H3.3 or H3.1 (*H3F3A*, *HIST1H3B*, and *HIST1H3C*) in approximately 78% of DIPGs, p53 mutations in 77% of cases, activating mutations in ACVR1 in 20–32%, and less frequently inactivating mutations in alpha-thalassemia/mental-retardation syndrome-X-linked (ATRX) [9–16]. Although the role of these mutations in gliomagenesis is still under intense investigation, recent studies have identified that the presence of H3F3A K27M mutations are associated with worse prognosis and decreased survival [9]. These histone mutants have been shown to globally decrease the repressive trimethylation of Lys27 on H3, resulting in changes in gene expression [17–19]. Other recently discovered alterations include amplification or mutations of components of the Receptor Tyrosine Kinase-Ras-PI3K signaling pathway, including gains in platelet-derived growth factor receptor (PDGFR-A) in up to 36% of patients [20–23]. Paugh et al. reported that 46% of DIPG with amplified and/or mutated PDGFRA also have inactivating mutations of TP53 [24].

In the United States, it remains uncommon for children with typical DIPGs to undergo stereotactic biopsy and many families elect to forgo autopsies, making human samples exceedingly rare for preclinical-targeted drug evaluation [25, 26]. Alternately, preclinical models of DIPG utilizing genetically engineered mice have been developed with histologic and immunophenotypic similarities to human DIPG samples, which help prioritize the translation of novel agents into clinical trials for children with DIPG [17, 27, 28]. As part of the Children’s Oncology Group CNS-DVL committee DIPG preclinical consortium, mouse model derived DIPG tumor cells were used in this study in an *in vitro* high-throughput drug screen to identify novel therapeutic agents with future potential to enter clinical trials. Through this screen, BMS-754807 was identified as a therapeutic agent of interest in DIPG.

BMS-754807 is a potent and reversible small molecule multi-kinase inhibitor of the insulin like growth factor-1 receptor (IGF-1R), Insulin receptor (IR), MET, TRKA, TRKB, AURKA, and AURKB among others [29]. IGF-1R signaling is active in several CNS tumors including medulloblastoma and glioblastoma [30, 31], and components of the pathway such as IGF-1R and IGF2 are amplified in up to 20% of DIPG [21]. Inhibiting the IGF-1R pathway has been shown to reduce tumor progression and halt metastasis formation in animal models of Ewing’s sarcoma and glioblastoma [32–34]. Targeting IGF-1R/IR (insulin receptor) signaling with BMS-754807 has also resulted in cell growth inhibition in several pediatric tumor types, *in vitro* and *in vivo* [35, 36]. In addition, MET, TRKA, TRKB, and AURKB have been implicated as potential therapeutic targets in DIPG [13, 21, 37].

Here we identify BMS-754807 as a cytotoxic agent in an *in vitro* drug screen using cells derived from genetically engineered mouse models of DIPG. We further evaluate its anti-tumor activity in a DIPG model driven by PDGF-B overexpression, H3.3 K27M overexpression, and p53 loss. *In vitro* BMS-754807 was found to have significant cytotoxic effects with an IC_50_ of 0.13 μM, and to significantly inhibit proliferation at concentrations of 1.5 μM. Interesting, serum-free cultures derived from the PDGF-B; H3.3K27M; p53 deficient model lack phosphorylated IGF-1R suggesting an IGF-1R independent mechanism for BMS-754807. *In vitro*, BMS-754807 inhibited AKT phosphorylation but did not inhibit PDGFR-A phosphorylation. *In vivo* we show that systemic administration of BMS-754807 to PDGF-B; H3.3K27M; p53 deficient DIPG-bearing mice does not significantly prolong survival; however, tissue drug levels were found to be lower than the calculated IC_50_ values. Together, these results identify BMS-754807 as a promising therapeutic agent in DIPG.

## Materials and Methods

### Mice

Nestin tv-a; p53^fl/fl^ (Np53^fl/fl^) mice were created by crossing Ntv-a mice with p53^fl/fl^ (C57BL/6J background) from Jackson Labs, as described in Barton et al [27]. GFAP tv-a; p53^fl/fl^ (Gp53^fl/fl^) mice were created by crossing Gtv-a mice [38] with p53 ^fl/fl^ (C57BL/6J background) from Jackson Labs. Nestin-tv-a;Ink4a-ARF-deficient mice have been previously described [28].

### DIPG tumor development

#### RCAS/tv-a mouse model of pediatric high-grade glioma

All animal studies were approved by the Duke University Institutional Animal Care and Use Committee. The RCAS/tv-a system was used to generate PDGF-B-driven DIPG mouse models with Ink4a-ARF-deficiency in Ntv-a mice [28], p53-deficiency in Ntv-a mice [27], p53-deficiency in Gtv-a mice, and p53-deficiency and overexpression of H3.3WT (PHC) or H3.3K27M (PKC) in Ntv-a mice [17]. DF1 (virus producing cells) cells were purchased from ATCC (Manassas, VA) and cultured in DMEM (ATCC) supplemented with 10% FBS, 2mM L-glutamine, 100 units/mL penicillin and 100μg/mL streptomycin, and incubated at 39°C and 5% CO_2_. Cells were transfected with RCAS plasmids (RCAS-PDGF-B, RCAS-Cre, RCAS-H3.3WT, or RCAS-H3.3K27M) using Fugene 6 or X-TremeGENE 9 (Roche) per the manufacturer’s instructions. Cells were used for injections after being passaged at least 6 times from the time of transfection. For the Gtv-a model, a mixture of DF1 cells (one μL, 10^5^ cells) expressing RCAS-PDGF-B and RCAS Cre at a 1:1 ratio were injected intracranially into the brainstem of postnatal day 3–5 GFAP-tv-a (Gtv-a); p53^fl/fl^ mice to generate brainstem glioma. For the PHC and PKC models, DF1 cells expressing RCAS-PDGF-B, RCAS-Cre and either the RCAS-H3.3 WT or the RCAS-H3.3 K27M at a 1:1:1 ratio were injected into the brainstem of postnatal day 3–5 nestin tv-a (Ntv-a) p53^fl/fl^ mice. Symptoms of brainstem gliomagenesis were notable starting at 3.5 weeks post injection including mild weight loss, decreased level of activity, mild ataxia, or mild head tilt. Mice were euthanized with CO_2_ when euthanasia end-points were reached (25% weight loss, lethargy) in accordance with Duke University IACUC protocol. Brain tissue from these animals was extracted, and either fixed in 10% neutral buffered formalin and paraffin embedded, or used to generate tumor cell lines (animals sacrificed between 29–45 days for PHC/PKC cell lines generation).

#### Neurosphere cell culture development

To generate murine DIPG neurospheres, whole tumors were extracted and incubated with 5mL papain solution (Papain 4.6 mg; EDTA 0.9 mg; Cysteine 0.9 mg; 5 mL Earls Balanced Salt Solution) plus 30μL DNase (10mg/mL, Sigma Aldrich) with trituration, followed by 2 mL of Ovomucoid solution (Ovomucoid stock solution: Ovomucoid protein 10 mg; 20 μL DNase) and centrifuged at 1100rpm. The pellet was then triturated in 0.5mL of ovomucoid, brought up with 5mL of Neurocult media (*Stem Cell Technologies, #05700*), centrifuged at 600rpm, and repeated 3 times. The cell pellet and aggregate supernatant were filtered and plated at a density of 500,000 cells per 25 cm^2^ flasks in serum free neurosphere media (per 50 mL total volume: 44.5 mL Neurocult basal media (*Stem Cell Technologies*); 10% proliferation supplement (*Stem Cell Technologies #05701);* Pen-strep 1% (*Invitrogen #15140–122*); Human basic FGF, 20ng/mL (*Invitrogen #13256–029)*; Human EGF, 10ng/mL (*Invitrogen #PHG0314)*; Heparin, 2μg/mL (*Stem Cell Technologies #07980)*. Cells were incubated at 37° C and split as required approximately once a week with Accutase (*Stem Cell Technologies*).

#### High-throughput drug screen

For this drug screen, we used four genetically distinct DIPG cell-lines (cultured in serum-free conditions) derived from the following genetically engineered mouse models of DIPG: 1) PDGF-B and p53 deficient DIPG tumor derived from a GFAP tv-a; p53 floxed mouse; 2) PDGF-B and p53 deficient DIPG tumor derived from a Nestin tv-a; p53 floxed mouse and 3–4) PDGF-B; Ink4-ARF deficient DIPG tumor that was infected with H3.3K27M or H3.3 WT *in vitro* as previously described [17]. Tumor cells were added to 384-well plates pre-plated with 60 known drugs in an increasing gradient of concentrations. Two separate versions of the drug plates were used in this study for a total of 83 unique drugs tested. Each drug was tested on at least two independent DIPG cell-lines derived from the four genetically distinct engineered mouse models of DIPG (alphabetical drug list in S1 Table). Drugs included in the screen were selected based on known DIPG target overexpression, and current availability for use in pediatric clinical testing. After 48 hours of incubation, CellTiter-Glo (*Promega #G7572*) cell viability assays were completed, and the IC_50_ for each drug was determined based on luminescence.

#### Cellular viability and proliferation assays

DIPG neurospheres were placed in 96-well plates in neurosphere media at a density of 50,000 cells/150μL media and incubated for 48 hours with BMS-754807 (*Active Biochemicals #A-1013*), or 0.1% DMSO control. A bromodeoxyuridine (BrdU) based cell proliferation ELISA assay kit (*Roche*) was used for proliferation and processed according to the manufacturer’s protocol. Absorbance was read using Molecular Devices Versa Max Tunable Microplate reader at 370nM and 492nM. Viability was measured with CellTiter-Glo Luminescent Cell Viability Assay *(Promega, # G7572)* per the manufacturer’s protocol; luminescence values were obtained using Turner Biosystems Modulus Microplate luminometer (*model 9300–062*). Statistical analyses were performed using Graph Pad Prism Software (LaJolla, CA, Version 5). Results reported are using one-way Mann-Whitney test. All values are given as mean +/- SE of at least 3 independent experiments, on a minimum of at least 2 different tumor cell lines.

#### Western blot analysis

Cells were cultured for 4 hours with BMS-754807 at 1 μM or 10 μM concentrations with or without IGF ligand (*Sigma Aldrich*, *I8779–50UG*), with matched controls. For stimulation experiments, IGF was added at 10nmol/L concentration for 15 minutes prior to cell collection. Cells were lysed in lysis buffer containing: 50mM Tris pH 7.5, 0.5M NaCl, 1% NP-40, 1% DOC, 0.1% SDS, 2mM EDTA, protease inhibitor cocktail and phosphatase inhibitor cocktail 2 (*Sigma*) and homogenized using a Glas-Col 099C-K54 homogenizer. Protein concentrations of total cell lysates or total tissue lysates were determined using BCA assay kit *(Pierce)*. Lysates were resolved by SDS-PAGE (*Invitrogen*) and transferred to nitrocellulose membranes (*Invitrogen #IB301002*).

After blocking in Odyssey Blocking Buffer (*Li-Cor Biosciences 927–40000*) with 0.2% Tween 20 (*Li-Cor Biosciences BP337–100*), membranes were incubated with antibodies for the following targets of interest: phospho-Akt^Ser473^ 1:2000 (#4060), Akt 1:1000 (#4685), pPDGFRA 1:1000 (#12022), PDGFRA 1:1000 (#3164) and actin 1:500 (# sc-1616). Membranes were incubated with the appropriate labeled secondary antibodies (*Li-Cor Biosciences*, *926–68024/926–32211*), and protein was visualized using Li-Cor Biosciences Odyssey Infrared Imaging system (*Li-Cor Biosciences*).

Blotting for phospho-IGF-1R 1:1000 (#3024), IGF-1R 1:1000 (#3018) required blocking in 5% non-fat milk and 0.1% TBS/Tween-20 at room temperature for one hour. Primary antibodies were prepared in 5% BSA/TBS-T and incubated overnight at 4°C. Secondary antibodies conjugated with horseradish peroxidase were prepared in blocking buffer and incubated at room temperature for one hour. Detection was performed with Amersham ECL Plus Detection kit according to the manufacturer’s protocol (GE Healthcare). All antibodies were purchased from Cell Signaling except Actin, which was purchased from Santa Cruz Biotech.

#### *In vivo* anti-tumor activity

All experiments were performed in accordance with the Institutional Animal Care and Use Committee and Institutional Biosafety Committee of Duke University approved protocol (A239-10-9 and A214-13-8). Ntv-a; p53^fl/fl^ mice were injected with DF1 cells expressing RCAS-PDGF-B + RCAS-Cre + RCAS-H3.3 K27M as described above. Three weeks post injection, mice were randomly assigned to one of two treatment groups: BMS-754807 or vehicle control by oral gavage. BMS-754807 was dissolved in a mixture of polyethylene glycol 400 and water (PEG_400_ 4:1 H_2_0) at 5.0 mg/mL. Mice were treated with 50mg/kg via oral gavage, once daily for three weeks or until euthanasia end-points were reached. Only mice that completed at least 5 doses of chemotherapy or 5 vehicle doses were included in the final analysis. Survival analysis was conducted as previously described [27]. Loss of 25% body weight, and lethargy was used as euthanasia criteria.

### Pharmacokinetic Analysis

#### Animal Therapy

Ntv-a; p53^fl/fl^ mice were injected with DF1 cells expressing RCAS-PDGF-B + RCAS-Cre + RCAS-H3.3 K27M as described above. Upon appearance of symptoms, mice were randomly assigned to one of two treatment groups: BMS-754807 or vehicle control by oral gavage. BMS-754807 was dissolved in a mixture of polyethylene glycol 400 and water (PEG_400_ 4:1 H_2_0) at 5.0 mg/mL as described above. Mice were treated with 50mg/kg via oral gavage, once daily for 3 doses and sacrificed 4 hours after the last dose.

#### Sample processing

Brain tissue was homogenized with 2 parts water. Homogenate from non-treated brain samples was used to prepare calibration samples in 0.096–60 ng/mL range. In 2-mL polypropylene screw-cap vial, 50 μL homogenate, 50 μL water, and 300 μL chloroform was added and vigorously mixed in Fast Prep (*Thermo-Savant*) agitator for 2 cycles at speed 4 for 20 sec. After centrifugation for 5 min at 14,000 g, upper (aqueous) layer and interface layer were aspirated out and 250 μL of chloroform layer transferred to glass tube and evaporated to dryness under nitrogen for 15 min. The dried material was dissolved with 30 μL acetonitrile followed by addition of 30 μL of 0.1% formic acid in water and mixture transferred into autosampler vial.

#### Liquid chromatography tandem-mass spectrometry (LC/MS/MS) assay

The analysis was performed on Shimadzu 20A series LC system coupled with Applied Biosciences/SCIEX API 4000 QTrap MS/MS. Column: Phenomenex, C18 4x3 mm, AJ0-4287 at 35°C. Solvent A: 0.1% formic acid in MS-grade water. Solvent B: MS-grade acetonitrile. Elution gradient at 1 mL/min: 0–1 min 20–90% B, 1–1.3 min 90% B, 1.3–1.4 min 90–20% B. Run time: 4min. Injection volume: 5 μL. The MS/MS parameters were optimized by infusion of 100 ng/mL of BMS-754807 at 10 μL/min. Mass transitions for quantification and confirmation (m/z): 462.0/321.8 and 462.0/197.8, respectively. DP: 71 V, EP: 10 V, ion-spray voltage: 4500 V, curtain gas: 30, ion-source gas1: 30, ion-source gas2: 25. Calibration curve used for quantification was linear (r = 0.9996) in the range from 0.096 ng/mL (LLOQ) to 60 ng/mL.

## Results

### High-throughput *in vitro* drug screen identifies BMS-754807 as a drug of interest for DIPG.

Secondary to the limited number of DIPG biopsy or autopsy tissue samples, there exists a significant backlog of novel therapeutics to potentially test in clinical trials. Therefore a large preclinical *in vitro* high-throughput screening of currently available therapeutics was needed to elucidate small molecule inhibitors with the potential to provide the greatest clinical yield. For this drug screen, primary cultures of DIPG neurospheres derived from four different genetically engineered mouse models of DIPG: 1) PDGF-B; p53 loss induced in GFAP-tv-a (Gtv-a); p53^fl/fl^ mice, 2) PDGF-B, p53 loss induced in Nestin-tv-a (Ntv-a); p53^fl/fl^ mice [27], and PDGF-B induced in Ink4a-ARF deficient mice [28] infected *in vitro* with either 3) H3.3K27M or 4) H3.3 WT, were added to one of two generations of drug plates with drug samples in an increasing gradient of concentrations for a total of 83 drug tested (S1 Table). Drugs included were selected based on known DIPG target overexpression, as well as drugs that are currently in other Children’s Oncology Group trials or have the potential to enter future clinical testing. Each drug was tested in at least two DIPG cell-lines from the four genetically engineered DIPG mouse models and then drugs were ranked by their average IC_50_ with the most 10 cytotoxic drugs listed in Table 1. Surprisingly, no PDGFR-A inhibitors were among these, consistent with the observations of others that PDGFR-A inhibitors are primarily cytostatic in gliomas driven by PDGF signaling [24]. BMS-754807 was the second most cytotoxic drug with an average IC_50_ of 160nM. As it is a multi-kinase inhibitor with several therapeutic targets of relevance to DIPG (Table 2), we chose to further investigate its efficacy by using the PDGF-B; H3.3K27M; p53 deficient DIPG model both *in vitro* and *in vivo*.

**Table 1:** Top Hits in a high-throughput *in vitro* drug screen across four distinct murine DIPG genotypes.

Drug	PDGF-B; p53 lossGFAP tv-aIC50 (μM)	PDGF-B; p53 lossNestin tv-aPlate 1IC50 (μM)	PDGF-B;p53 loss; Nestin tv-a; Plate 2IC50 (μM)	PDGF-B;Cdkn2a null;H3.3 WTIC50 (μM)	PDGF-B;Cdkn2a null H3.3K27MIC50 (μM)	Average IC50 (μM) across all cell-lines
Dinaciclib	N/A	N/A	0.01	0.03	0.02	0.02
**BMS754807**	**0.01**	**0.1**	**0.04**	**0.48**	**0.15**	**0.16**
AUY922	N/A	N/A	0.18	0.27	0.41	0.29
Carfilzomib	0.14	0.83	0.18	0.16	0.23	0.31
BMS387032	0.22	0.5	0.61	1.55	0.33	0.64
SAHA	0.16	1.33	0.3	0.92	0.53	0.65
Entinostat	N/A	N/A	1.11	0.63	1.02	0.92
Trichostatin	0.31	1.12	1.75	0.53	1.08	0.96
Panobinostat	0.01	2.65	0.19	1.59	0.57	1.00
Fenretinide	N/A	N/A	1.69	0.37	1.26	1.11

The top 10 hits in the *in vitro* drug screen in each of four distinct genetically engineered DIPG cell lines: 1) PDGF-B; p53 deficient DIPG cells induced in Gtv-a; p53^fl/fl^ mice. 2) PDGF-B; p53 deficient DIPG cells induced in Ntv-a; p53^fl/fl^ mice, 3) PDGFB; Ink4a-ARF deficient DIPG cells infected with H3.3 WT *in vitro*, 4) PDGFB; Ink4a-ARF deficient DIPG cells infected with H3.3 K27M *in vitro*.

**Table 2:** Select Glioma Relevant Targets of BMS-754807^a^.

Target	Kd (nM) BMS-754807
IGF-1R	1.8
Insulin R	1.7
MET	5.6
ALK	5.7
TRKA	7.4
TRKB	4.1
AURKA	9
AURKB	25
JAK2	347
CDK2	795

BMS-754807 targets with their respective dissociation constants (K_d_)

^a^ Table is derived from Carboni *et al*. [29].

### *In vitro*, BMS-754807 reduces proliferation and viability of DIPG neurospheres in a dose-dependent manner

**Fig 1:**
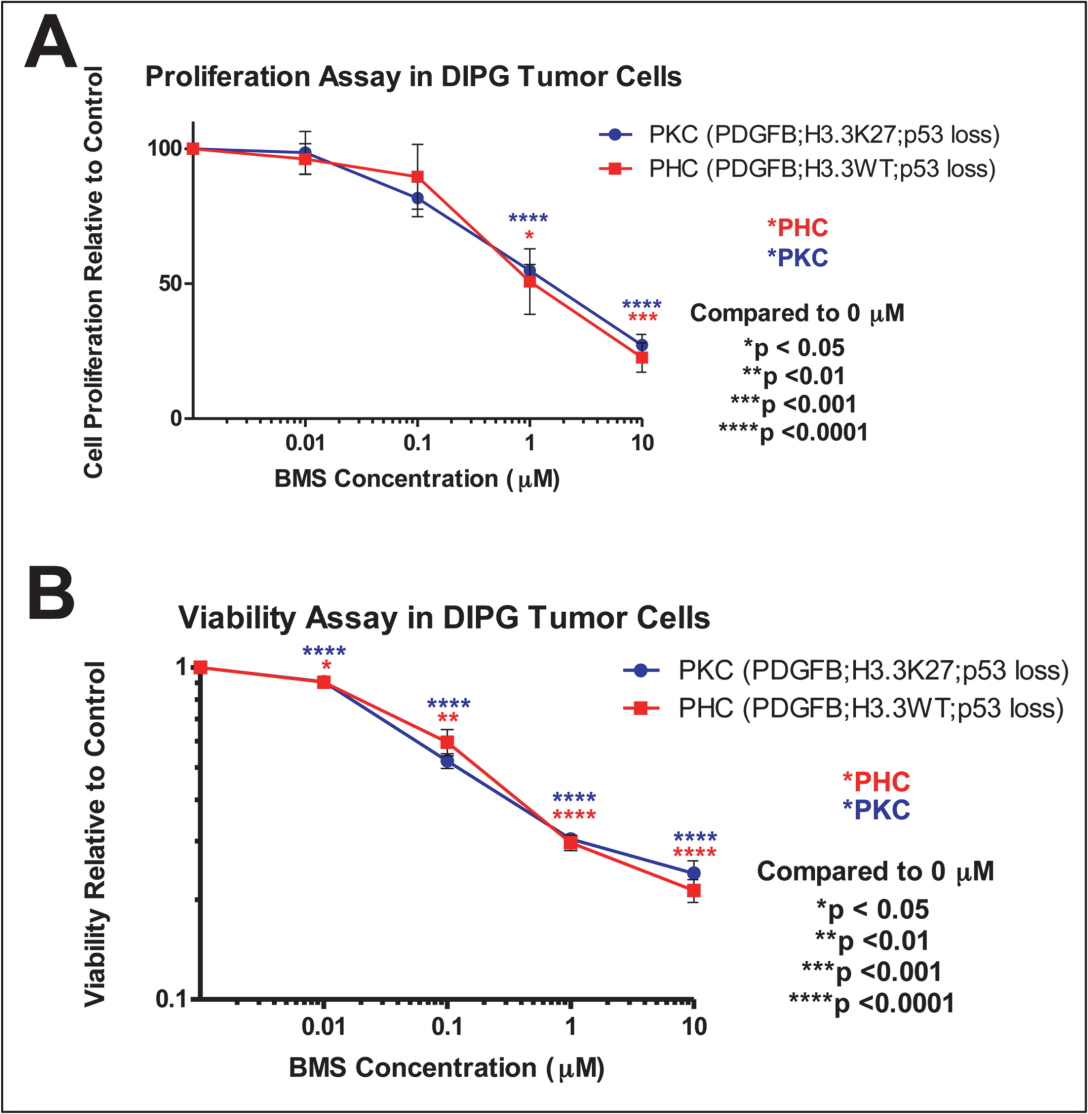
BMS-754807 inhibits proliferation and reduces viability of DIPG neurospheres in a dose-dependent manner. Cells were isolated from mouse DIPG induced by PDGF-B, H3.3K27M, and p53 loss (PKC) or PDGF-B; H3.3WT, and p53 loss (PHC), cultured *in vitro* as neurospheres, treated with BMS-754807 or vehicle at the indicated doses for 48 hours, and assayed for (A) cell proliferation using BrdU incorporation and (B) cell viability using Cell Titer Glo. All statistics were compared to 0 μm.

We first tested the effects of BMS-754807 in tumor neurospheres derived from mouse DIPG driven by PDGF-B overexpression, p53 loss, and either the wild type H3.3 histone (PHC) or the mutant H3.3-K27M histone (PKC). BMS-754807 significantly inhibited proliferation as demonstrated by BrdU assay with an IC_50_ of 1.5 μM (Fig. 1A). Viability assay also showed significant reduction in the survival of DIPG neurospheres at drug concentrations of 0.01 μM and higher, with an IC_50_ of 0.13 μM (Fig. 1B). These results suggest that the effects of BMS-754807 on proliferation and survival may be independent of histone H3.3 status, as both the wild type- and K27M-expressing DIPG cells were equally sensitive to treatment.

**Fig 2:**
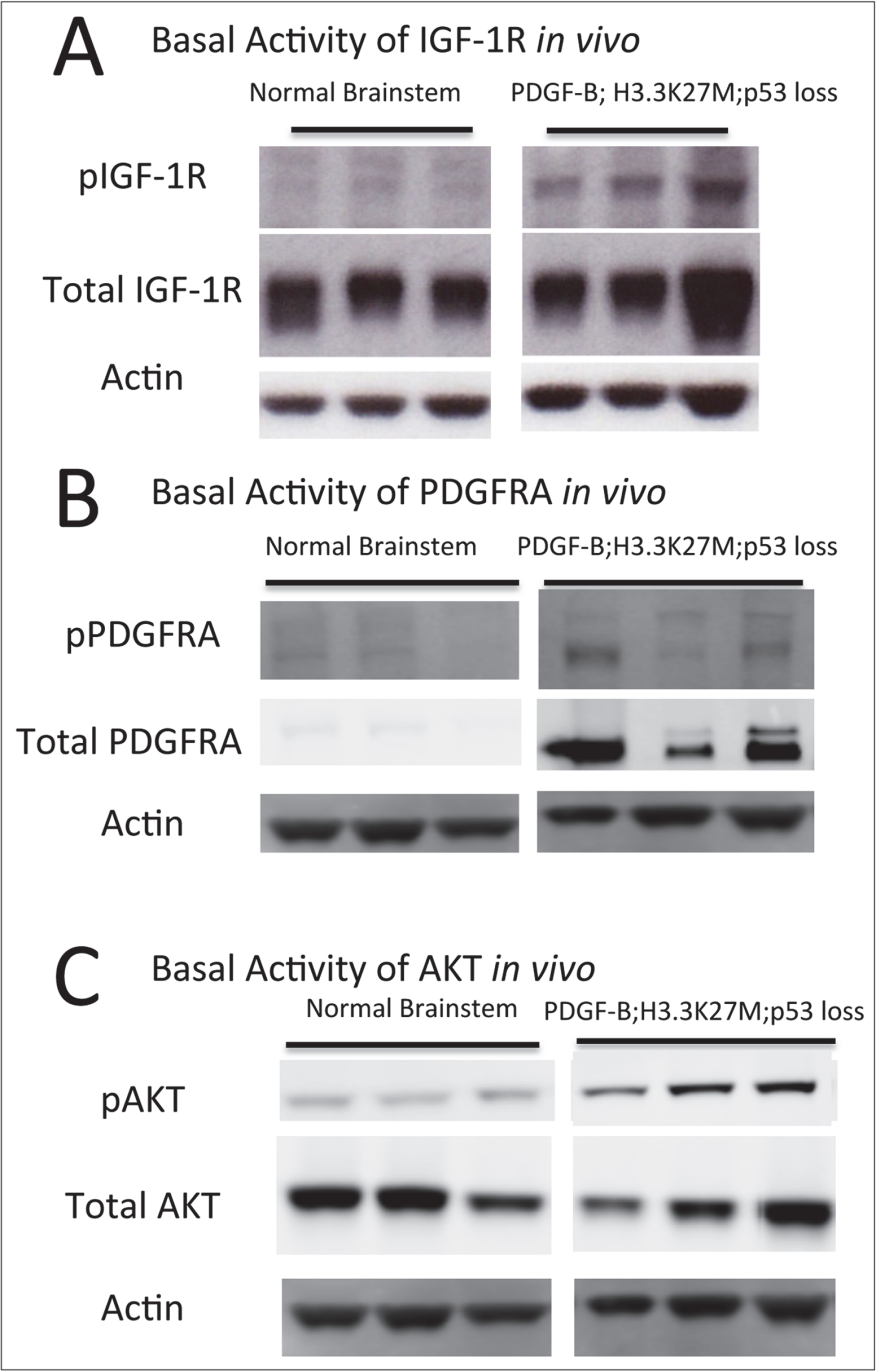
Phosphorylated and total levels of IGF-1R, PDGFRA and AKT in murine DIPG. Western blot for (A) pIGF-IR (95 kDa) and total IGF-1R (95 kDa), (B) pPDGFRA (195 kDa) and total PDGFRA (195 kDa), and (C) pAKT (60 kDa) and total AKT (60 kDa) in normal mouse brainstem (n = 3) and mouse DIPG driven by PDGF-B, H3.3-K27M, and p53 loss (n = 3). Actin (43 kDa) is shown as a loading control.

As one of the potential targets of BMS-754807 is IGF-1R, we performed a western blot on whole tumor lysates from our mouse model and observed only a slight increase in the levels of phosphorylated IGF-1R in all tumors tested as compared to normal brainstem (Fig. 2A). As our model is driven by PDGF signaling we also chose to examine basal expression of PDGFRA and downstream AKT. We observed increased levels of phosphorylated PDGFRA, total PDGFRA (Fig. 2B), and phosphorylated AKT (Fig. 2C) in the tumor samples but no significant changes in total AKT levels.

**Fig 3:**
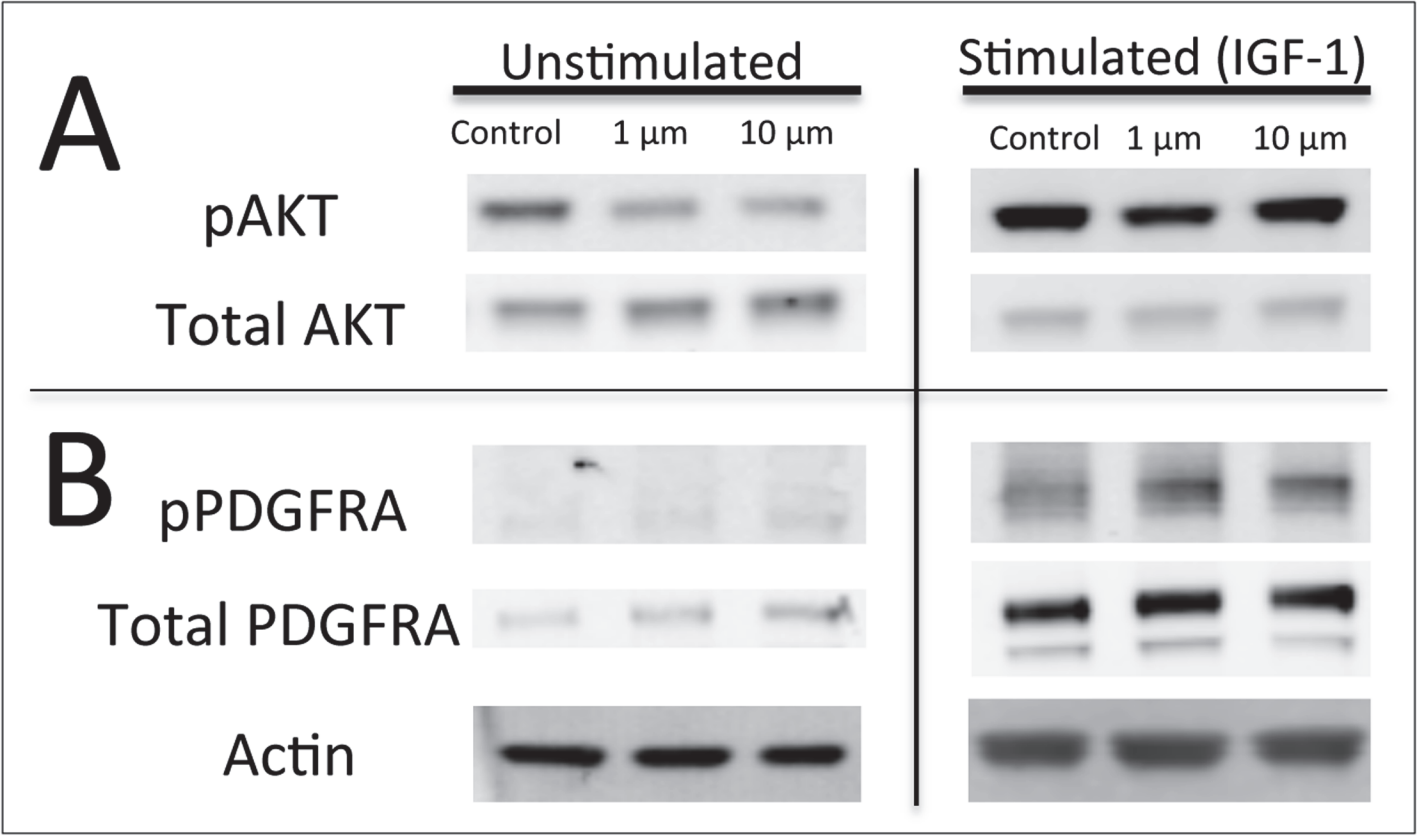
BMS-754807 decreases AKT phosphorylation in DIPG cell-lines cultured in serum-free media. Western blot for (A) pAKT (Ser473, 60 kDa) and total AKT (60 kDa), (B) pPDGFRA (195 kDa) and total PDGFRA (195 kDa) after four hours of treatment with BMS-754807 with or without IGF ligand stimulation for 15 minutes in DIPG cell lines induced by PDGF-B, H3.3K27M, and p53 loss. A representative blot from three independent experiments is shown. Actin (43 kDa) is shown as a loading control.

To investigate the mechanism of BMS-754807 in this DIPG model, we treated neurospheres derived from mouse PDGF-B; H3.3K27M; p53-deficient tumors with 1μM and 10μM of drug in the presence or absence of IGF ligand. There was no phosphorylated IGF-1R in the neurospheres, with or without IGF-1 ligand stimulation (S1 Fig.). We did however note phosphorylated AKT (Serine 473), which is downstream of several known BMS-754807 targets such as MET, TRKA, TRKB, IR (Fig. 3A). Phosphorylated AKT was inhibited after 4 hours of BMS-754807 treatment *in vitro*, both with and without stimulation with IGF-1 ligand with the exception of the 10μM dose in the stimulated cells (Fig. 3A). In addition, we investigated the possibility that BMS-754807 is targeting PDGFRA (which is activated in our mouse model, as shown in Fig. 2B). However, we observed no reduction in phosphorylated PDGFRA expression with BMS-754807 treatment (Fig. 3B).

### BMS-754807 does not significantly extend survival *in vivo*

**Fig 4:**
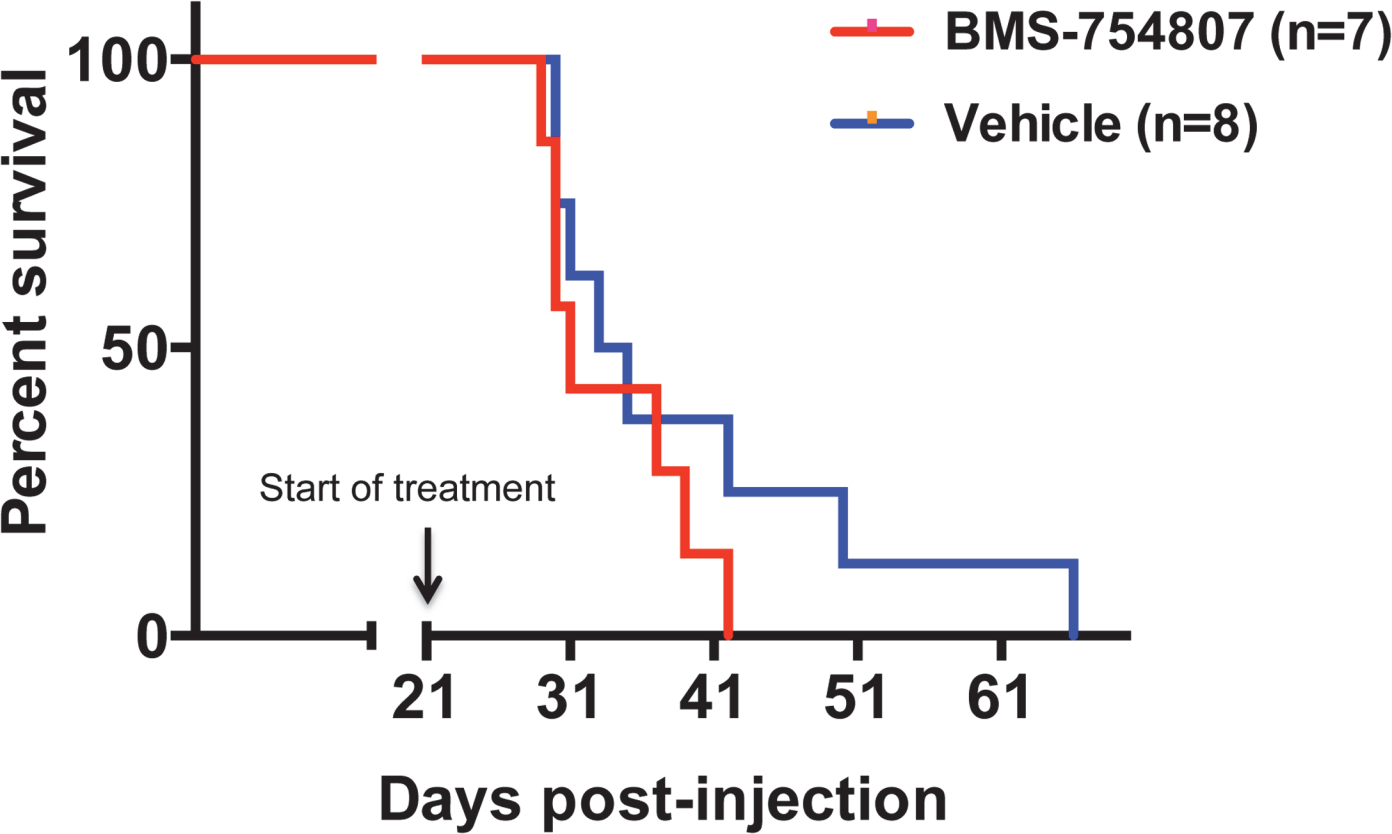
BMS-754807 does not prolong survival *in vivo*. Nestin tv-a (Ntv-a); p53^fl/fl^ mice were injected with DF1 cells expressing RCAS-PDGF-B + RCAS-Cre + RCAS-H3.3 K27M. Three weeks post injection, mice were randomly assigned to one of two treatment groups: BMS-754807 or vehicle control. Mice were treated with 50mg/kg via oral gavage, once daily for 21 doses. Mice were monitored daily and sacrificed when mice were moribund and/or lost 25% body weight. Only those mice that received at least 5 doses of drug or vehicle were included in the final analysis. Kaplan-Meier survival curves demonstrated no significant survival benefit with BMS-754807 therapy. The median survival for the vehicle alone group was 34 days vs. 31 days for the BMS-754807 alone group (p = 0.26 by Log-Rank Test).

**Fig 5:**
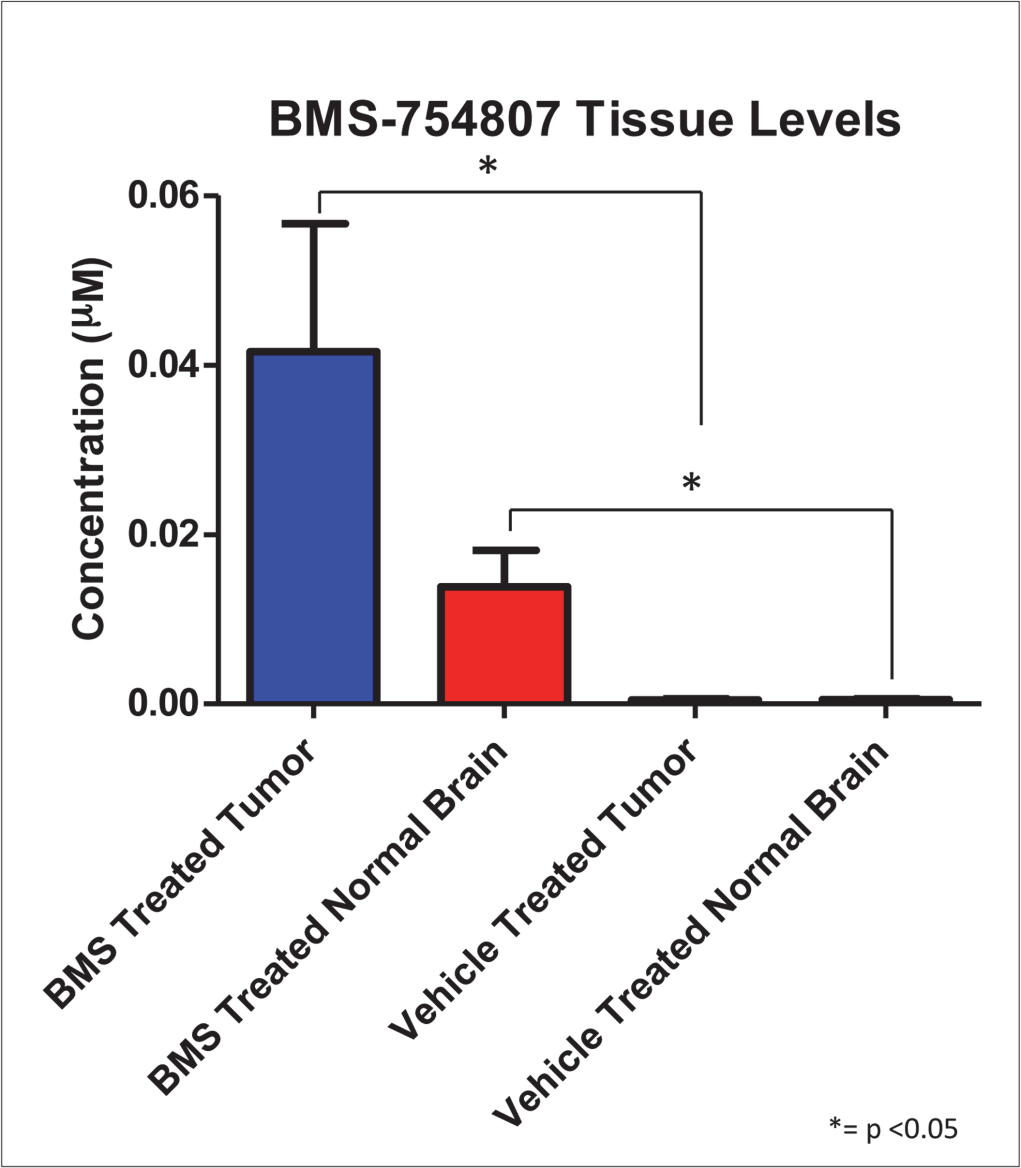
BMS-754807 has limited drug delivery to the tumor in the brainstem. Nestin-Tv-a;p53^fl/fl^ mice were injected with DF1 cells expressing RCAS-PDGF-B, RCAS-Cre, and RCAS-H3.3-K27M. Upon appearance of symptoms, mice were treated with either BMS-754807 or vehicle for 3 doses, and sacrificed at 4 hours after the last dose. Tissue concentrations of BMS-754807 were determined by liquid chromatography tandem-mass spectrometry (LC/MS/MS). There was a significant difference between BMS-754807 treated tumor lysates compared to vehicle treated tumor lysates (p = 0.0357 by Mann-Whitney) and between BMS-754807 treated normal brain lysates as compared to vehicle treated normal brain lysates (p = 0.0357 by Mann-Whitney).

Due to its efficacy *in vitro*, we were interested to determine if BMS-754807 can significantly prolong survival of DIPG-bearing mice as a single agent *in vivo*. Nestin expressing brainstem progenitors of neonatal Ntv-a; p53^fl/fl^ mice were infected with PDGFB, H3.3 K27M, and Cre at postnatal day 3–5. At 3 weeks post injection, mice were randomized to treatment with either BMS-754807 (50 mg/kg oral gavage) or vehicle once a day for three weeks (or until euthanasia criteria were met) and monitored for symptoms of tumor formation. No significant survival benefit was observed with BMS-754807 treatment, as the median survival for the vehicle group was 34 days vs. 31 days for the BMS-754807 group (n = 7 for BMS-754807 and n = 8 for vehicle; p = 0.26 by Log-Rank) (Fig. 4). To investigate achievable intra-tumor drug concentrations, tumor-bearing mice were treated with BMS-754807 for 3 days at the same dose used in the survival study and sacrificed 4 hours after the last dose. Based on liquid chromatography tandem-mass spectrometry (LC/MS/MS), we observed significant levels of drug present within the brain tissue of these animals compared with vehicle-treated mice, however the levels were well below the previously determined IC_50_ values observed *in vitro* (Fig. 5). This suggests that limited drug delivery of BMS-754807 to the tumor in the brainstem may explain why daily BMS-754807 treatment to DIPG-bearing mice did not significantly prolong survival.

## Discussion

An *in vitro* high-throughput drug screen conducted in collaboration with the Children’s Oncology Group CNS-DVL committee preclinical consortium identified BMS-754807 as a drug of interest using cell-lines derived from our genetically engineered mouse models of DIPG. In the current study, we evaluated the efficacy of BMS-754807, a known multi-kinase inhibitor whose targets include IGF-1R, Insulin-R, MET, ALK, TRKA, TRKB, AURKA and AURKB both *in vitro* and *in vivo* using the PDGF-B; H3.3K27M; p53 deficient DIPG model. Advantages of this genetically engineered mouse model include the ability to generate tumors with well-defined genetic alterations in the brainstem of immunocompetent mice. The tumors developed in this model are infiltrative and invade the surrounding normal brain similar to human DIPG lesions. However, genetically engineered mouse models such as this one may not harbor the full complement of genetic alterations found in the human disease and so preclinical drug screenings should also be performed in parallel with human tumor derived cells or mouse models. Another potential limitation of this model is that PDGF signaling is activated by the PDGF-B ligand while in the human disease it is more often activated by the PDGFRA receptor through amplification or mutation [22].

As BMS-754807 is primarily known as an IGF-1R inhibitor, we initially focused on determining whether IGF-1R signaling was present in this model. We observed that our DIPG mouse model driven by PDGF-B, H3.3-K27M, and p53 loss has only slightly increased levels of IGF-1R phosphorylation relative to normal brainstem. Serum-free cultures derived from these tumors did not harbor any discernable IGF-1R phosphorylation with or without IGF-1 stimulation suggesting that the mechanism of BMS-754807 in this model is independent of IGF-1R. Serum-free cultures were sensitive to treatment with BMS-754807, showing reductions in survival, proliferation, and AKT activation. The cytotoxic mechanism of BMS-754807 in this model is independent of PDGFR-A as there was no observed change to PDGFR-A phosphorylation in response to BMS-754807. In addition, we observed that the sensitivity of DIPG cells to BMS-754807 might be independent of H3.3-K27M status.

The identities of the active targets of BMS-754807 in our mouse model are currently unknown. Although BMS-754807 most potently inhibits IGF-1R and IR activation [29] it is a relatively non-specific drug, and the reduction in AKT phosphorylation as well as the cytotoxic effects seen in this study may be due to inhibition of one or more of its other targets including MET, ALK, TRKA, TRKB, AURKA, AURKB, JAK2, CDK2 [29]. Indeed, it has previously been shown that AURKB may be a potential therapeutic target in DIPG [37]. Our *in vitro* experiments were conducted in serum-free conditions which selects for stem-like tumor cells with the addition of EGF and FGF [39]. One caveat of our results is that these conditions may not precisely replicate the *in vivo* intra-tumoral microenvironment of the brainstem in children with DIPGs. However, stem-cell conditions have been shown to be more clinically relevant than adherent serum-based conditions, promoting more similar gene expression and activated signaling pathways as compared to the *in vivo* tumor milieu [39].

Despite significant cytotoxic effects of BMS-754807 *in vitro*, treatment of DIPG-bearing mice *in vivo* with BMS-754807 did not result in a survival benefit as compared to vehicle-treated mice. It is worth noting that the *in vivo* dose chosen of 50mg/kg/day in our study (as well as lower doses) has been demonstrated to be efficacious in several solid tumor mouse models such as breast cancer, prostate cancer, and rhabdomyosarcoma [29, 35, 36, 40, 41]. Analysis of tumor tissue harvested after short-term BMS-754807 treatment indicated that the level of drug present in the tumor tissue was well below the IC_50_, suggesting inadequate drug delivery to the tumor, as a potential explanation for the lack of efficacy *in vivo*. BMS-754807 was, however, well tolerated by mice undergoing treatment, leaving the possibility of combining this drug with additional agents or other delivery mechanisms like convection enhanced delivery to allow for its improved delivery to the tumor in the brainstem.

## Conclusion

Here we use a genetically engineered mouse model of DIPG that includes the H3.3K27M mutation as a novel platform through which *in vitro* and *in vivo* evaluations can help prioritize drug therapies for clinical trials for children with DIPG. These findings demonstrate that BMS-754807, a potent multi-kinase inhibitor, can significantly reduce both viability and proliferation of DIPG tumor cells at doses of 0.1 μM and higher *in vitro*. We also demonstrate BMS-754807-mediated inhibition of AKT. The efficacy of BMS-754807 *in vitro* did not translate to a survival benefit *in vivo* potentially due to the limited drug delivery to the brainstem. If given with additional agents or delivered locally to increase its delivery to the tumor, it has the potential to be efficacious *in vivo*. These results are promising and imply that BMS-754807 is a potential therapeutic agent for DIPG and future experiments aim to identify additional therapies that may synergize with BMS-754807.

## Supporting Information

S1 Fig There is no discernable IGF-1R phosphorylation in PDGF-B; H3.3K27M; p53 deficient neurospheres *in vitro*. Western blot analysis for pIGF-1R (95 kDa) and total IGF-1R (95 kDa) in DIPG cell lines driven by PDGF-B, H3.3K27M, and p53 loss. Cells were treated with BMS-754807 for 4 hours at the indicated doses with or without IGF ligand stimulation for 15 minutes. Actin (43 kDa) is shown as a loading control. A representative blot from three independent experiments is shown.
(TIF)

S1 Table Results of the High-Throughput *In vitro* Drug Screen. 83 drugs were screened across 4 distinct DIPG genotypes (each drug was evaluated in at least two DIPG cell-lines).
(XLSX)
