# Supplementary figures and images for: A High-Throughput In Vitro Drug Screen in a Genetically Engineered Mouse Model of Diffuse Intrinsic Pontine Glioma Identifies BMS-754807 as a Promising Therapeutic Agent
Source: PLoS One. 2015 Mar 6;10(3):e0118926. doi: 10.1371/journal.pone.0118926 (PMC4352073; doi:10.1371/journal.pone.0118926)

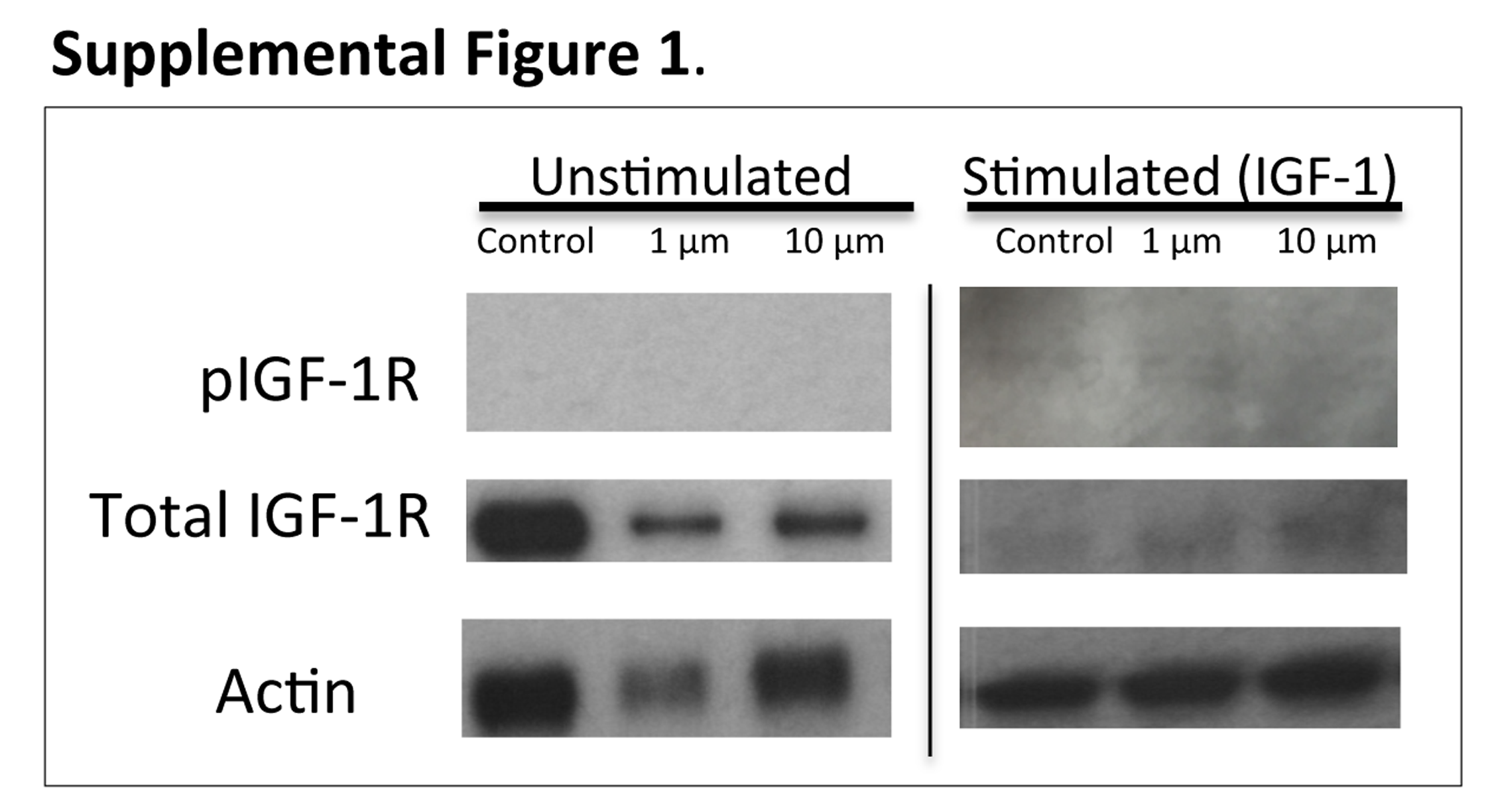

Supplement: S1 Fig — Western blot analysis for pIGF-1R (95 kDa) and total IGF-1R (95 kDa) in DIPG cell lines driven by PDGF-B, H3.3K27M, and p53 loss. Cells were treated with BMS-754807 for 4 hours at the indicated doses with or without IGF ligand stimulation for 15 minutes. Actin (43 kDa) is shown as a loading control. A representative blot from three independent experiments is shown. (TIF) [file pone.0118926.s001.tif]
